# Treatment of Dysentery

**Published:** 1851-09

**Authors:** 


### EDITORIAL DEPARTMENT.

*Treatment of Dysentery.* — The following letter has been received since the *original communications* for this No. were in type. As the disease to which it relates prevails especially at the present season of the year, we introduce the article here, rather than defer it for the next issue, subjoining a few remarks:

DEAR SIR: — The discrepancy of standard authors on the treatment of dysentery, and its alarming fatality when occurring as an epidemic, alike show the science of medicine hitherto at fault, in mastering this dreadful scourge.

Seeing the valuable article, by Dr. Tullis, in the April number of your very excellent journal, on the *morphine* treatment of dysentery, has induced me to make some additional remarks, which I think of the utmost importance in the treatment of this disease.

The remedy of the first importance, and first to be employed, is *bloodletting,* a remedy sadly and extensively neglected. In acute *enteritis,* it is our sheet anchor: and that practitioner who would neglect it, in a severe case, would at once be pronounced guilty of bad practice, and he *ought* to be considered equally culpable if he neglect it in a severe case of *dysentery.* It is nothing less than a most violent inflammation of the mucous membrane of the colon and rectum — differing only in its *seat,* from acute enteritis — and bears the lancet with equally good effect. The objection so frequently urged, that the typhoid tendency in this disease forbids venesection, is wholly invalid; — and ought not to be worth any more than if urged in acute enteritis. Understand me:— these remarks on bloodletting relate to *acute* dysentery in its *first* stage; and if freely employed at the onset, and followed by the after treatment I shall prescribe, I will venture to predict that *never* will an uncomplicated case of dysentery run into a typhoid condition. The *rule* will be, recovery is *certain.* To be *effectual,* it must be employed *early;* and then it is *indeed effectual.* The remedy next to be employed, is, — a mild, yet *thorough* and *complete* evacuation of the stomach and bowels. The reason for this is plain. The highly inflamed colon and rectum being acutely sensitive, every particle of fecal matter from the upper bowels, so soon as it reaches the inflamed parts, induces the most painful contractions of the sensitive bowel; and if bloodletting be immediately followed by *anodynes*, without first unloading the stomach and upper bowels, their contents will be continually dribbling over the inflamed and denuded surface of the lower bowel, to the exquisite torture of the patient, and the alarming aggravation of the disease. That the *smallest particle* of matter from above, will produce the most distressing tormina and tenesmus, when it comes in contact with the inflamed parts below, is clearly shown in the fact, that a patient, in a dozen or more ineffectual efforts at stool, will not be able to void a half ounce of fecal matter. Therefore the indication is plain to let *nothing* pass over the inflamed surface, except what is absolutely *unavoidable.* Now the contents of the upper bowels must of *necessity* pass over it, for you cannot hold them there by anodynes or astringents, until you can, by any means, cure the inflammation below. Therefore, good sense would teach, at *once*, to clear the stomach and upper bowels, and not let their contents, be *days* in passing the inflamed parts below, to their constant irritation. Observation and experience have taught us, that a *free* and *full* evacuation of the upper bowels, gives the patient infinitely less pain, than the continued irritation of smaller quantities; and the reason is probably this: in the first case, the inflamed bowel is excited to contraction by an emollient substance, which in turn presents a tender and yielding resistance,— while in the second case, it is excited by the smallest quantity, and left to contract upon *itself*— the most intolerable of all pains to be endured.

Having reduced the severity of the inflammation by bloodletting, and *thoroughly* cleared the stomach and bowels, I *then* adopt the *morphine* or anodyne treatment, with an *absolute* diet. *Diet* is of the *utmost importance* at this stage. It matters not of how soothing a character that may be, which is taken into the stomach, if it come in contact with the inflamed bowel, it will endure the most intolerable griping; and there is nothing more certain, than the *aggravation*, and *extension* of the disease, are the result of the painful *contraction* of the sensitive bowel upon *itself*, rather than of the *character* of what passes over it. Therefore, the most soothing mucilages should be exhibited, as food and drink, and in the smallest possible quantities—and then be retained in the stomach and upper bowels, until removed by the absorbents — and to meet this last indication, we use anodyne and astringents,— especially the morphine, combined with tannin and prepared chalk, — and in no case have they had any other effect than to *reduce* the *fever*, and *frequency* of stools, and give the suffering patient quiet and rest. And I frequently continue to exhibit them for four or six days, before giving any laxative, and have had no unpleasant symptom follow. In connection with the means already named, I *always* use warm fomentations to the bowels, and sometimes the cups.

The above treatment I believe to be in accordance with good sense, and the true pathology of the disease; and a uniform success, in a variety of cases, of every grade of severity, has given me unlimited confidence in it. I would further add, that my invaluable friend Dr. G. Bogart, of Union Corners, *has* been, and is *now*, treating the disease upon the above-named principles, with the most pleasing success. Within the last three days, I have had the pleasure of seeing him extricate a darling child, but a year old, from the jaws of death, by the prompt use of the lancet, and other means I have named.

The case was one of alarming severity, ushered in by convulsions, the highest febrile excitement, and the most distressing tormina, every fifteen or twenty minutes followed by stools of bloody mucus. After bleeding and evacuating the bowels, the child was put upon the anodyne treatment, and in less than twenty-four hours, the little sufferer was fairly convalescent; and last night I had the pleasure of seeing him sit at table with his mother, and cry for a *mutton bone.* We now have the disease prevailing here epidemically, and the success of the above treatment has been entire, thus far.

One word in relation to Dr. Tullis' morphine treatment. He concludes by saying — "In all ordinary cases of dysentery, suitable doses of morphine, after each evacuation, with rest and abstinence, will effect a speedy cure;" and he proposes to make further observations on the "*severer*" forms of dysentery. Now I can but believe, that even in "ordinary" cases of dysentery, to exhibit morphine so freely, previous to venesection, and with the stomach and bowels loaded, if it be *good practice*, is not the *best.* It certainly is crippling the influence of a most valuable remedy: for if it be so effectual, with the contents of the upper bowels draining over the inflamed surface below, how *much more* effectual must it be, when it has undisputed sway of the alimentary canal. As to the severer forms of the disease, it *must* be ineffectual; while the treatment I have prescribed, is most applicable to the severest forms. And in *no* case of well marked dysentery, can there be the least hazard in placing your patient in the upright posture, and bleeding until you affect the pulse.

I communicate this for publication in your valuable journal, if you deem it of sufficient worth, and have not a supply of more important matter on hand. Yours truly,

Tuscarora, Liv. Co., Aug. 21, 1851. F. MORSE, M. D.

*Remarks.* — The foregoing communication would have been more satisfactory had the author specified the amount of his experience, that is, the number of cases treated, with an analysis of symptoms sufficiently to show the severity of the disease. Few affections are more variable, as respects gravity, and the characters entering into its natural history than dysentery. In its ordinary sporadic form, the inflammation limited to the lower part of the large intestine, and not of great intensity, it is by no means attended with much danger in the larger proportion of cases. This, doubtless, explains, in a measure, the apparent success of different, and opposing methods of practice. As a general remark, it is only when it occurs as an epidemic that it is attended by a large fatality; but different epidemics differ widely in this respect, as well as in the diversified features which they assume.

Of bloodletting in dysentery, we cannot speak from personal experience. There is considerable discrepancy of opinion with practitioners on this, as on other points pertaining to the treatment. That this remedy is adapted to all cases, or every epidemic, we cannot believe, but it is not improbable that they are at fault who repudiate its employment in all instances of this disease. Perhaps it is unduly neglected by many practitioners. Bloodletting in various diseases is practiced to much less extent by judicious physicians now, than formerly, and it may be that, from the tendency to antithetical extremes, this potent therapeutical measure sometimes fails to receive, at the present time, an adequate appreciation of its merits. If this be so, it is possible that dysentery is one of the diseases in the treatment of which the remedy might enter with advantage to a greater extent than is generally supposed. Careful observations with reference to this important subject are desirable; and also with reference to the discrimination of the cases in which the remedy is called for, from those in which it is not required, and consequently hurtful. As respects cathartics, we concur with our correspondent in their propriety at the commencement of the disease, for the reasons stated. By effecting a pretty thorough discharge of the intestinal contents, they prepare for the use of opiates. This object does not call for a frequent repetition of cathartics. After a satisfactory operation therefrom, they need not ordinarily be repeated for several days. If we mistake not, it is a common error to persist too long in the use of cathartic remedies, or to prescribe them, with too short intervals, in this disease. In this way the ulterior object is counteracted. The advantage of cathartics does not consist in their immediate effects on the inflamed intestine. Limited to these effects, they would be improper remedies. They do good by relieving the bowels of their fecal contents, so that they may subsequently be kept in a state of quiescence, favorable for the resolution of the inflammation. Quietude of the inflamed part is the leading indication for management, in so far as the intestinal affection is concerned. And for this end, after proper cathartics, opiate remedies are the most efficient. These remedies, to use the common metaphor, constitute, in our opinion, the sheet anchor in the treatment of dysentery. To be effective, they must be given for effect. In other words we are to be guided in the quantity given by the results produced. Opiates may frequently be prescribed with great freedom in dysentery without any symptoms of narcotism. We have been surprised at the doses borne by patients with this disease. For example, in a case recently under observation, within the space of eight and a half hours, three and a half grains of morphia were given, in doses of half a grain at intervals of one and two hours, together with two enemas, one containing a drachm, and the other nearly two drachms of laudanum. No symptoms of narcotism were occasioned. We do by no means say that the same quantity, in the same period, would be proper, or safe in many cases. The doses are to be regulated by circumstances, and always repeated at intervals sufficient for the practitioner to observe the degree of narcotic influence, so as to avoid danger from this source. The precept to be enforced is, that, to secure quietude to the inflamed intestine, opiates are to be employed to the extent required for that object, proceeding cautiously, watching the effect of doses given at intervals, graduating the amount to the degree of tolerance, and the favorable impression on the disease.

Our correspondent does not allude to mercurials in the treatment of dysentery. He is evidently not of the class of *liver* doctors who consider that *hepatic obstruction* is the fundamental pathological element involved in an inflammation of the large intestine, and prescribe calomel, as a matter of course, *to excite the secretion of bile!* We have not time nor space to dwell on this point, but will give, in conclusion, the following extract from *Sketches of the Diseases of India*, by Mr. Annesley. We are indebted for the extract to the late work on Therapeutics, by Prof. Thomas D. Mitchell, of Philadelphia. We publish it for the benefit of any of our readers whose minds may not be wholly divested of the *hepatic pathology* of the past generation, and who fancy that the term *bilious*, as commonly used, has a definite signification. Mr. Annesley performed a series of experiments for the express purpose of ascertaining the true operation of calomel, "and these experiments presented uniform results, viz., that whilst the stomach and duodenum of dogs that had taken large doses of this preparation were much paler and less vascular than in ordinary circumstances, the colon and rectum, from the cæcum to the verge of the anus, were most acutely inflamed, thereby explaining the results of clinical observation — namely, that, although large doses of calomel calm those symptoms usually caused by increased vascular action, or inflammation of the mucous surface of the stomach and duodenum, they lower the vital energy of these important organs, and occasion tenesmus, griping pains in the course of the colon, mucous or bloody stools, hemorrhoids; and, if persisted in, many more of the symptoms of dysentery, or even structural change of the colon and rectum. I am confident that dysentery becomes chronic; that an occasional indigestion lapses into a constant dyspepsia; and that habitual constipation often passes into strictures of the rectum, and hemorrhoids into fistulæ, from the frequent exhibition of large doses of this medicine. Ingenuity cannot possibly devise a more successful method of converting a healthy person into a confirmed invalid, of destroying many of the comforts of existence, and of occasioning hypochondriasis and melancholy, than the practice of prescribing large doses of calomel on every trifling occasion, or when the bowels require gentle assistance; or because the patient erroneously supposes himself to be *bilious*, or is told so by those who should know better. The unfortunate word '*bilious*' is the scape-goat of the ignorant."

